# Supplementary material for: Mutational Spectrum and Clinical Features of Patients with LOXHD1 Variants Identified in an 8074 Hearing Loss Patient Cohort
Source: Genes (Basel). 2019 Sep 23;10(10):735. doi: 10.3390/genes10100735 (PMC6826470; doi:10.3390/genes10100735)
Supplement: Supplementary file 1 [file genes-10-00735-s001.pdf]

Table S1. 68 Deafness-causative genes

| No | Locus Symbol | Gene Symbol | Gene ID | Transcript Variant |
|---|---|---|---|---|
| 1 | DFNA1 | *DIAPH1* | NM_005219 | NM_001079812 |
| 2 | DFNA2 | *KCNQ4* | NM_004700 | NM_172163 |
| 3 | DFNA2 | *GJB3* | NM_024009 | NM_001005752 |
| 4 | DFNA3 | *GJB6* | NM_006783 | NM_001110219<br>NM_001110220<br>NM_001110221 |
| 5 | DFNA4 | *MYH14* | NM_024729 | NM_001145809<br>NM_001077186 |
| 6 | DFNA5 | *DFNA5* | NM_004403 | NM_001127454<br>NM_001127453 |
| 7 | DFNA6/14/38 | *WFS1* | NM_006005 | NM_001145853 |
| 8 | DFNA8/12/DFNB21 | *TECTA* | NM_005422 | - |
| 9 | DFNA9/31 | *COCH* | NM_004086 | NM_001135058 |
| 10 | DFNA10 | *EYA4* | NM_004100 | NM_172103<br>NM_172105 |
| 11 | DFNA11/DFNB2/USH1B | *MYO7A* | NM_000260 | NM_001127179<br>NM_001127180 |
| 12 | DFNA13/DFNB53/STL3 | *COL11A2* | NM_080680 | NM_080679<br>NM_080681<br>NM_001163771 |
| 13 | DFNA15 | *POU4F3* | NM_002700 | - |
| 14 | DFNA17 | *MYH9* | NM_002473 | - |
| 15 | DFNA20/DFNB26 | *ACTG1* | NM_001614 | NM_001199954 |
| 16 | DFNA22/DFNB37 | *MYO6* | NM_004999 | - |
| 17 | DFNA25 | *SLC17A8* | NM_139319 | NM_001145288 |
| 18 | DFNA28 | *GRHL2* | NM_024915 | - |
| 19 | DFNA36/DFNB7/11 | *TMC1* | NM_138691 | - |
| 20 | DFNA40 | *CRYM* | NM_001888 | NM_001014444 |
| 21 | DFNA44 | *CCDC50* | NM_178335 | NM_174908 |
| 22 | DFNA48 | *MYO1A* | NM_005379 | - |
| 23 | DFNA50 | *MIRN96* | | |
| 24 | DFNA51 | *TJP2* | NM_001170414 | NM_004817<br>NM_201629<br>NM_001170630<br>NM_001170415<br>NM_001170416 |
| 25 | DFNA64 | *DIABLO* | NM_019887 | NM_138929 |
| 26 | | *CEACAM16* | NM_001039213 | - |
| 27 | DFNB1/DFNA3 | *GJB2* | NM_004004 | - |
| 28 | DFNB3 | *MYO15A* | NM_016239 | - |
| 29 | DFNB4/Pendred | *SLC26A4* | NM_000441 | - |
| 30 | DFNB6 | *TMIE* | NM_147196 | - |

| | | | |
|---|---|---|---|
| 31 DFNB8/10 | *TMPRSS3* | NM_024022 | NM_032405 |
| 32 DFNB9 | *OTOF* | NM_194248 | NM_194322<br>NM_194323<br>NM_004802 |
| 33 DFNB12 | *CDH23* | NM_022124 | NM_001171930<br>NM_001171931<br>NM_001171932<br>NM_001171933<br>NM_001171934<br>NM_001171936<br>NM_052836 |
| 34 DFNB15/72/95 | *GIPC3* | NM_133261 | – |
| 35 DFNB16 | *STRC* | NM_153700 | – |
| 36 DFNB18/USH1C | *USH1C* | NM_153676 | NM_005709 |
| 37 DFNB22 | *OTOA* | NM_144672 | NM_001161683<br>NM_170664 |
| 38 DFNB23/USH1F | *PCDH15* | NM_033056 | NM_001142763<br>NM_001142764<br>NM_001142765<br>NM_001142766<br>NM_001142767<br>NM_001142769<br>NM_001142770<br>NM_001142771<br>NM_001142772<br>NM_001142773 |
| 39 DFNB24 | *RDX* | NM_002906 | – |
| 40 DFNB25 | *GRXCR1* | NM_001080476 | – |
| 41 DFNB28 | *TRIOBP* | NM_007032 | NM_138632<br>NM_001039141 |
| 42 DFNB29 | *CLDN14* | NM_144492 | NM_001146077<br>NM_001146078<br>NM_001146079<br>NM_012130 |
| 43 DFNB30 | *MYO3A* | NM_017433 | – |
| 44 DFNB31/USH2D | *WHRN* | NM_015404 | NM_001083885<br>NM_001173425 |
| 45 DFNB35 | *ESRRB* | NM_004452 | – |
| 46 DFNB36 | *ESPN* | NM_031475 | – |
| 47 DFNB39 | *HGF* | NM_000601 | NM_001010931<br>NM_001010932<br>NM_001010933<br>NM_001010934 |
| 48 DFNB42 | *ILDR1* | NM_001199799 | NM_001199800<br>NM_175924 |
| 49 DFNB48 | *CIB2* | NM_006383 | NM_001271888<br>NM_001271889 |
| 50 DFNB49 | *MARVELD2* | NM_001038603 | NM_001244734 |

| | | | | |
|---|---|---|---|---|
| 51 | DFNB59 | *DFNB59* | NM_001042702 | – |
| 52 | DFNB61 | *SLC26A5* | NM_206883 | NM_001167962<br>NM_206884<br>NM_206885<br>NM_198999 |
| 53 | DFNB63 | *LRTOMT* | NM_001145307 | NM_001145308<br>NM_001205138<br>NM_145309 |
| 54 | DFNB66/67 | *LHFPL5* | NM_182548 | – |
| 55 | DFNB70 | *PNPT1* | NM_033109 | – |
| 56 | DFNB74 | *MSRB3* | NM_198080 | NM_001031679<br>NM_001193460<br>NM_001193461 |
| 57 | DFNB77 | *LOXHD1* | NM_144612 | NM_001173129<br>NM_001145472<br>NM_001145473 |
| 58 | DFNB79 | *TPRN* | NM_001128228 | – |
| 59 | DFNB82 | *GPSM2* | NM_013296 | – |
| 60 | DFNB84 | *PTPRQ* | NM_001145026 | – |
| 61 | DFNB89 | *KARS* | NM_005548 | NM_001130089 |
| 62 | DFNB91 | *SERPINB6* | NM_004568 | NM_001195291 |
| 63 | DFNB93 | *CABP2* | NM_016366 | – |
| 64 | USH2A | *USH2A* | NM_007123 | NM_206933 |
| 65 | DFNX1 | *PRPS1* | NM_002764 | NM_001204402 |
| 66 | DFNX2 | *POU3F4* | NM_000307 | – |
| 67 | DFNX4 | *SMPX* | NM_014332 | – |
| 68 | DFNX6 | *COL4A6* | NM_001847 | NM_033641 |
